# NON-INFERIORITY OF DIGITALLY ASSISTED OUTPATIENT REHABILITATION IN PATIENTS WITH BACK PAIN: 12-MONTH FOLLOW-UP OF A RANDOMIZED CONTROLLED TRIAL

**DOI:** 10.2340/jrm.v58.44366

**Published:** 2026-05-20

**Authors:** Richard ALBERS, Stella LEMKE, David FAUSER, Franziska SCHÄFFER, Sebastian KNAPP, Gert KRISCHAK, Matthias BETHGE

**Affiliations:** 1 University of Lübeck, Institute of Social Medicine and Epidemiology, Lübeck; 2 ZAR Regensburg, Regensburg; 3 GOREHA GmbH, Berlin; 4 Nanz Medico GmbH & Co. KG, Stuttgart, Germany

**Keywords:** telerehabilitation, digital health, telemedicine, back pain, randomized controlled trial

## Abstract

**Objective:**

To examine the non-inferiority of digitally assisted, multimodal rehabilitation that utilizes a digital version of a standardized back school (intervention group) compared with a rehabilitation program applying the back school conventionally in person (control group).

**Design:**

Nonblinded, randomized, controlled, non-inferiority trial with 12-month follow-up after the end of rehabilitation.

**Subjects/Patients:**

Adults aged 18–65 years with back pain.

**Methods:**

The back school was part of a 3-week rehabilitation program along with other treatments in accordance with the therapy standards. Eight outpatient rehabilitation centers conducted the rehabilitation program. Self-reported pain self-efficacy (10–60 points) was the primary outcome. Non-inferiority was established if the lower limit of the one-sided 95% confidence interval was greater than –4 points for pain self-efficacy.

**Results:**

A total of 157 participants (55.3%) completed the 12-month follow-up. Multiple imputation of missing data allowed for inclusion of 270 participants in the analysis (intervention group: *n* = 127, control group: *n* = 143). Our primary adjusted intention- to-treat analysis demonstrated that digitally assisted rehabilitation was non-inferior to conventional rehabilitation at the 12-month follow-up (b = 0.48; 95% CI = –3.09 to ∞).

**Conclusion:**

This study supports the hypothesis that digitally assisted rehabilitation is a viable alternative to in-person rehabilitation for patients with back pain.

## LAY ABSTRACT

This study looked at whether a partly digital back pain rehabilitation program could work just as well as a traditional, fully in-person version. Adults with back pain took part in a 3-week outpatient rehabilitation program focused on exercises and education. One group completed the entire program in person, while the other group completed the exact same program with some parts, such as the educational content, delivered digitally. After 1 year, both groups showed similar improvements in how confident they felt about managing their pain. The partly digitally delivered program was found to be just as effective as the in-person program. This matters because it shows that adding digital elements to rehabilitation can maintain quality while offering greater flexibility. Making parts of the treatment digital can save time, reduce travel, and help people stick to their rehabilitation – especially those with busy schedules or limited access to clinics.

With a high prevalence of around 15.6% among adults in Germany (1), back pain is strongly associated with work disability (2) and high costs (3, 4). Both the causes and effects of back pain are biopsychosocial, such as pain, physical limitations, depression, social life, and inability to work, and interact with each other (5, 6). Rehabilitation programs that incorporate a multidisciplinary biopsychosocial approach are therefore recommended in back pain treatment guidelines worldwide and have been shown to reduce pain and disability in people with chronic low back pain (5, 7, 8).

Telerehabilitative interventions can increase access to therapy for patients, e.g., because long travel distances are no longer necessary or therapy can be more conveniently planned into everyday life (9). Telerehabilitative multidisciplinary biopsychosocial treatments could therefore improve treatment availability for people with back pain if such programs are comparably effective. To our knowledge, however, there are only 2 studies that have compared a remote app-based intervention with an in-person intervention, which used a multimodal (10, 11) and multidisciplinary (10) approach, and demonstrated similar outcomes in both groups (10, 11) or even better outcomes in favour of telerehabilitation (10). Neither of the 2 studies reported long-term effects, as did most other studies on digital interventions in rehabilitative settings. Long-term follow-up studies are, however, particularly important when health issues are recurrent or chronic and lifestyle changes need to be maintained over an extended period to allow health changes to become visible (12, 13).

In Germany, rehabilitation for back pain is typically conducted as a multidisciplinary 3-week program. A key component of these 3-week programs is an evidence-based, multimodal, and multidisciplinary back school. The back school consists of structured patient education (i.e., seminars), supervised exercising, and self-management training to increase physical activity in everyday life and change cognitive patterns that lead to chronic pain (14–16). In preparation for our study, we digitized this back school and transformed the conventional 3-week in-person program into a digitally assisted rehabilitation program (intervention group). In the digitally assisted rehabilitation program, the digitized back school was integrated into the multidisciplinary 3-week rehabilitation program, which – except for the back school component – was delivered in person at the outpatient rehabilitation centre. The digitized back school was completed at home. We aimed to demonstrate that patients who complete the digitally assisted rehabilitation program achieve pain self-efficacy comparable to those who participate in the conventional in-person rehabilitation program. In our previous publication, we presented the study results at the end of rehabilitation and at the 3-month follow-up (17). In this publication, we present the long-term effects (12-month follow-up) of our study.

## METHODS

### Trial design

To investigate whether patients completing the digitally assisted rehabilitation program achieve pain self-efficacy comparable to patients participating in a conventional in-person rehabilitation program, we conducted a randomized controlled trial (18). In this paper, we report the findings of the final 12-month follow-up. Findings on the short-term non-inferiority at the end of rehabilitation and at the 3-month follow-up can be found elsewhere (17). Patients were recruited at 8 outpatient rehabilitation centers in 7 German cities (Berlin, Bielefeld, Frankfurt am Main, Jena, Munich, Paderborn, and Regensburg). The intervention group participated in the digital back school remotely. The digital components were provided by Caspar Health, an e-health company that offers digital prevention, rehabilitation, and aftercare.

This study adhered to the principles outlined in the Declaration of Helsinki. Approval for the research was granted by the Ethics Committee of the University of Lübeck (21–462) on 25 March 2022, prior to its commencement. A more detailed overview can be found in the study protocol (18). We used the Consolidated Standards of Reporting Trials (CONSORT) and the extension for randomized non-inferiority and equivalence trials when drafting the manuscript (19, 20).

### Setting and participants

In Germany, patients with back pain typically enter outpatient rehabilitation through a physician’s referral. A prerequisite for the referral is a confirmed medical need, which is determined by the treating physician (e.g., general practitioner) and documented in a rehabilitation application submitted by individuals to the relevant health insurance provider or pension insurance agency. Upon approval, patients are assigned to a certified rehabilitation facility. Outpatient rehabilitation is generally carried out close to the patient’s place of residence. The eligibility of potential study participants was assessed by study assistants employed at the respective outpatient rehabilitation centers at the start of the rehabilitation program. Recruitment was subsequently initiated.

Patients between the ages of 18 and 65 who experienced back pain (ICD-10 M50-M54, post-acute and acute rehabilitation) were eligible to participate in the study. Individuals without reliable internet access, a suitable electronic device for app-related videos, or a functional camera for communication were excluded. Patients not proficient in German were also excluded.

### Intervention group

The intervention group was provided with a digitally assisted rehabilitation program, including a digital back school. The standardized back school was created by the German Pension Insurance and is grounded in both the health action process approach and the fear-avoidance beliefs model (14, 15, 21). A randomized controlled trial demonstrated the effectiveness of this standardized back school. However, implementation took place in an inpatient setting and was carried out in person (14). In this study, the Caspar application was used for the digital implementation and remote delivery of a digital version of the standardized back school. An overview of the back school in the intervention group is presented in Table SI in accordance with the Template for Intervention Description and Replication (TIDieR) checklist (22) and the TIDieR-Telehealth checklist (23). Further information on the intervention can be found in the study protocol (18).

### Control group

The control group received a conventional rehabilitation program, including the conventional version of the standardized back school as in-person meetings (16). An overview of the back school in the control group is presented in Table SII following the TIDieR checklist (22). Further information on the intervention can be found in the study protocol (18).

### Both groups

The full 3-week rehabilitation program included, in addition to the back school, various other treatments. These treatments were in line with the therapy standards set by the German Pension Insurance for individuals with chronic back pain and primarily included exercise therapy, physiotherapy, and psychosocial interventions (16). The therapy standards for chronic back pain stipulate that all patients complete at least 46.1 h of therapy throughout the rehabilitation period, provided that all intended treatment components are clinically appropriate. The objective of the program was to enhance the patients’ functional abilities and to facilitate or restore their participation in work and daily activities.

### Outcomes

*Primary outcome.* The primary outcome was pain self-efficacy as reported by the participants and was assessed using the German version of the Pain Self-Efficacy Questionnaire (PSEQ, German: Fragebogen zur Erfassung der schmerzspezifischen Selbstwirksamkeit [FESS]) (24, 25). The score ranges from 10–60. Higher scores indicate better pain self-efficacy (24).

*Secondary outcomes.* The secondary outcomes included general health (measured by the Copenhagen Psychosocial Questionnaire [COPSOQ], 0–10 points) (26), mental health, functional capacity, and pain (assessed using the Indicators of Rehabilitation Status [IRES-24] questionnaire, 0–10 points) (27), and cognitive and behavioural pain management (28). The latter encompassed subscales such as action-oriented coping, cognitive restructuring, subjective coping competence, mental distraction, counter-activities, and relaxation, which were evaluated using the Questionnaire for the Assessment of Pain Coping ([FESV], 4–24 points) (28).

Additional secondary outcomes consisted of knowledge concerning back pain and treatment (0–50 points), self-efficacy in applying the learned knowledge (0–20 points), and electronic health literacy (measured by the E Health Literacy Scale, [eHEALS], 8-40 points) (29). Three self-created questions on self-informing behaviour, exercise adherence after the end of rehabilitation, and knowledge adherence after the end of rehabilitation were each evaluated categorically and transformed into binary variables (at least once per week vs less than once per week).

Additional secondary outcomes related to work included work ability, which was evaluated using 3 questions from the Work Ability Index (30, 31). The first 2 questions inquired about the current capacity to perform physical and mental work (2–10 points). The third question required participants to rate their overall work ability on a scale from 0 to 10 points (32, 33). Additionally, the assessment included current sickness absence, length of sickness absence (in weeks), and current employment status (yes or no).

Only participants in the intervention group gave feedback on the digital program. We evaluated the usability of the Caspar application using the System Usability Scale (0–100 points, with scores of 70 or above considered acceptable) (34, 35) and collected an overall rating of the Caspar application (1 = very good, 2 = good, 3 = satisfactory, 4 = adequate, 5 = inadequate) (36). Scores from the System Usability Scale were further categorized into 5 ranges: 0–59, 60–69, 70–79, 80–89, and 90–100 (35).

### Sample size

We established that a difference of 4 points in pain self-efficacy represents the smallest meaningful difference between the intervention and control group (24, 37). Our non-inferiority analysis required a minimum sample size of 242 participants (one-sided error: 5%; power: 90%).

### Randomization and blinding

Randomized allocation was performed on a one-to-one ratio, with the principal investigator from the University of Lübeck responsible for generating all randomization sequences using Stata/SE version 16.1 (StataCorp LLC, College Station, TX, USA). Forty assignments were randomly grouped into blocks of 4 and 8. This was done for each outpatient rehabilitation centre individually. The University of Lübeck supplied the rehabilitation centers with 40 sealed, non-transparent envelopes containing only the study identification number and the information about the group allocation. After the participants had given informed consent, each envelope, numbered from 1 to 40, was handed out consecutively to the participants by a study assistant in each outpatient rehabilitation centre. The content of the envelope was concealed from all study personnel, with the exception of the principal investigator, who was not involved in participant recruitment. At each outpatient rehabilitation centre, a study assistant was responsible for enrolling participants and recording their group allocation in a study list (Microsoft Excel; Microsoft Corp, Redmond, WA, USA) using the assigned identification number. Both groups used the same consent forms. Participants, treatment staff, and the assessors of the data were not blinded. Additional information is provided elsewhere (18).

### Statistical analysis

We calculated descriptive statistics to determine the sample characteristics. To assess baseline group differences, we used a two-sample *t*-test for continuous variables and the Pearson χ^2^ test for categorical variables.

The confidence interval (CI) approach was used to assess the primary hypothesis of non-inferiority (19, 20). If the lower limit of the one-sided 95% CI exceeded –4 points, non-inferiority of the digitally assisted rehabilitation was assumed. The treatment effect estimate was adjusted for both the baseline score of the dependent variable and the rehabilitation centre. A linear regression model was used to estimate the difference in pain self-efficacy and its CI. Sex, education level, age, motivational self-efficacy, and electronic health literacy were considered as potential moderators of the treatment effect on the primary outcome. The interaction effects were assessed using linear regression models, along with the corresponding *p*-values.

Linear or logistic regression was conducted to estimate differences between groups for secondary continuous and binary outcomes. Regression coefficients or odds ratios were calculated, along with their 95% CIs and *p*-values. Superiority of the intervention group was tested for all secondary outcomes to identify potential group differences that, if non-inferiority was established, could help determine a preference for digitally assisted rehabilitation or conventional rehabilitation in person (38).

For the primary adjusted intention-to-treat analysis, we addressed missing data by performing multiple imputations, generating 20 independent datasets (39, 40). The imputation model incorporated covariates with no missing values, including sex, age, native German language skills, group allocation, and outpatient rehabilitation centre (location), as well as covariates with missing values, such as pain self-efficacy (primary outcome). The latter showed 41.9% of missing values at the 12-month follow-up. The parameter estimates were subsequently combined according to Rubin’s rules (41).

In the sensitivity analyses, we calculated several estimates: a non-adjusted intention-to-treat estimate, an adjusted per-protocol analysis estimate, and an estimate for a complete-case analysis. In the per-protocol analysis, we included only participants from both groups who attended at least 6 of the 7 back school modules, and we adjusted for the baseline score of the dependent variable and the rehabilitation centre. In the complete case analysis, we included only participants with complete data on all variables included in the model and adjusted for the baseline score of the dependent variable and the rehabilitation centre.

*P*-values < 0.05 were considered statistically significant, with all hypothesis tests conducted as two-tailed, except for the primary outcome. Any participant who withdrew consent had their data removed from the analysis. Stata/SE version 16.1 was used to perform all analyses.

## RESULTS

### Participant flow and recruitment

**Fig. 1:**
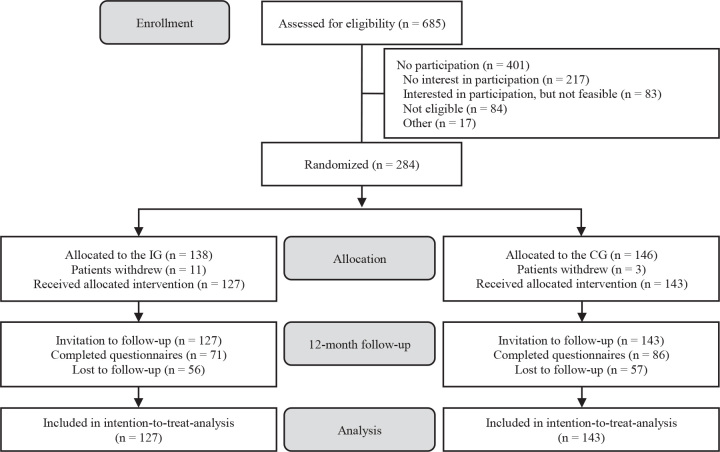
Flow of participants. CG: control group; IG: intervention group.

Between 5 April 2022, and 31 January 2023, study assistants at the outpatient rehabilitation centers assessed 685 patients for enrolment, of whom 284 were randomly assigned to either the intervention group (*n* = 138) or the control group (*n* = 146). Recruitment ended once an adequate sample size was achieved. A total of 14 patients were excluded after withdrawing consent and requesting the removal of their data. Twelve months after the end of rehabilitation, 157 participants completed the follow-up questionnaire (55.3%) (intervention group: *n* = 71; control group: *n* = 86). In our intention-to-treat analyses, we included 270 participants, using multiple imputation to replace missing values. The participant flow is presented in Fig. 1 (42).

### Sample characteristics

The average age of participants was 46.8 years; 52.6% were women, 55.8% reported an intermediate level of education (levels 3 to 4 of the International Standard Classification of Education [ISCED-11]), and 36.6% reported a high level of education (levels 5 to 8 of the ISCED-11). Table I presents the sample characteristics for both the intervention and control groups. The 2 groups showed comparable levels of health and functional impairment at baseline, with no significant differences found.

**Table I:** Sample characteristics of the participants

Item	IG (*n* = 127)		CG (*n* = 143)		*p*-value*
	*n*	M (SD) or %	*n*	M (SD) or %	
Sociodemographic data					
Age in years	127	46.5 (10.4)	143	47.0 (10.4)	0.667
Sex					0.662
Female	65	51.2%	77	53.8%	
Male	62	48.8%	66	46.2%	
Native German speaker					0.721
Yes	111	87.4%	127	88.8%	
No	16	12.6%	16	11.2%	
Partnership					0.809
Yes	94	78.3%	109	79.6%	
No	26	21.7%	28	20.4%	
Level of education					0.480
Low	12	9.6%	8	5.7%	
Medium	69	55.2%	79	56.4%	
High	44	35.2%	53	37.9%	
Primary outcome					
Pain self-efficacy (10–60)	120	38.6 (11.4)	136	37.3 (10.0)	0.313
Secondary outcomes					
Current health status (0–10)	125	4.9 (1.9)	142	4.8 (1.8)	0.722
Mental health (0–10)	125	4.9 (2.3)	141	5.2 (2.3)	0.385
Functional capacity (0–10)	125	4.4 (2.1)	138	4.1 (2.0)	0.230
Pain (0–10)^a^	125	3.2 (1.8)	141	2.8 (1.6)	0.073
Action-oriented coping (4–24)	123	16.3 (4.8)	135	16.0 (4.8)	0.654
Cognitive restructuring (4–24)	122	13.3 (4.7)	133	12.5 (4.3)	0.186
Subjective coping competence (4–24)	124	16.7 (4.0)	136	16.3 (4.2)	0.415
Mental distraction (4–24)	122	11.0 (4.4)	138	11.3 (4.8)	0.584
Counter-activities (4–24)	124	13.3 (4.6)	135	12.6 (4.6)	0.229
Relaxation (4–24)	122	11.4 (4.7)	137	10.8 (4.9)	0.273
Disorder and treatment knowledge (0–50)	121	23.7 (12.0)	135	24.7 (11.9)	0.500
Self-efficacy in practising knowledge (0–20)	124	10.3 (4.4)	134	10.3 (4.6)	0.879
Electronic health literacy (8–40)	124	29.2 (6.6)	135	29.4 (5.9)	0.883
Self-informing behaviour					0.807
At least once a week	41	32.8%	43	31.4%	
Less than once a week	84	67.2%	94	68.6%	
Employment					0.629
Yes	113	90.4%	124	88.6%	
No	12	9.6%	16	11.4%	
Off work due to sickness absence					0.537
Yes	65	52.0%	77	55.8%	
No	60	48.0%	61	44.2%	
Work ability in relation to work demands (2–10)	122	5.9 (1.7)	140	5.9 (2.0)	0.975
Self-rated work ability (0–10)	120	4.4 (2.3)	142	4.5 (2.6)	0.612
Sickness absence (weeks)	126	9.7 (9.1)	138	9.3 (9.0)	0.705

Deviations in the number of cases in the rows are due to missing values.

CG: control group; IG: intervention group; M: mean.

a High values represent low pain.

* Two-sample *t*-test or Pearson χ^2^ test.

### Delivered dose of treatment, usability, and use of the Caspar application

A difference of 2.2 h in treatment dose was observed, with the intervention group receiving 69.9 h and the control group 67.7 h. In accordance with the therapy standards of the German Pension Insurance for patients with chronic back pain, both groups completed a minimum of 46.1 therapy hours (16). Assessment of the Caspar application at the end of rehabilitation revealed acceptable system usability (mean = 75.7, standard deviation = 14.4). Approximately 76% of the participants in the intervention group rated the Caspar application as at least acceptable (> 70 points). Overall, 80.8% of the intervention group assessed the Caspar application as good or better. A more detailed overview of the delivered dose of treatment, usability, and the use of the Caspar application, as well as information on the number of completed back school modules per group and further outcomes, can be found elsewhere (17).

### Primary outcome

**Fig. 2:**
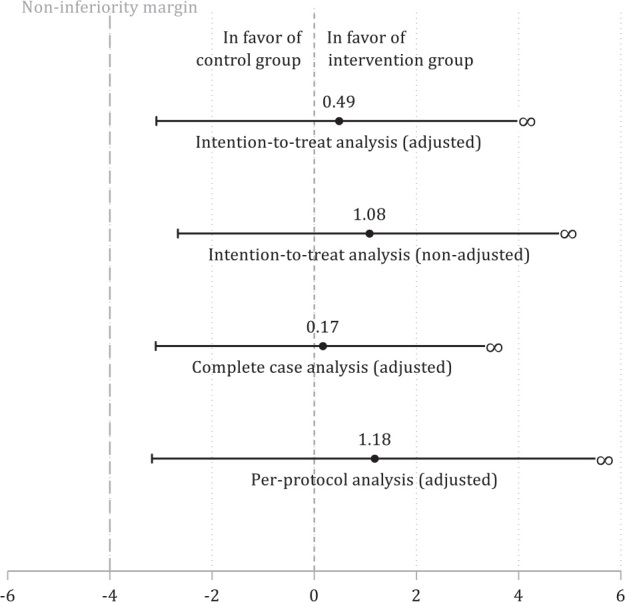
Testing non-inferiority of the digitally assisted rehabilitation using one-sided 95% confidence intervals at the 12-month follow-up.

At the 12-month follow-up, pain self-efficacy was slightly in favour of the intervention group. The lower limit of the 95% CI for the intervention group surpassed the non-inferiority margin of –4 points, demonstrating the intervention group’s non-inferiority. Non-inferiority was also confirmed in the non-adjusted intention-to-treat analysis, as well as by the adjusted complete-case analysis and the adjusted per-protocol analysis (Table II, Fig. 2).

**Table II:** Pain self-efficacy at the 12-month follow-up

Factor	IG			CG			b	One-sided 95% CI
	*n*	Predicted values	SE	*n*	Predicted values	SE		
12-month follow-up (adjusted ITTA)	127	43.83	1.35	143	43.35	1.27	0.48	–3.09 to ∞
12-month follow-up (non-adjusted ITTA)	127	44.15	1.39	143	43.07	1.34	1.08	–2.66 to ∞
12-month follow-up (adjusted CCA)	63	44.98	1.21	81	44.81	1.11	0.16	–3.10 to ∞
12-month follow-up (adjusted PPA)	82	44.05	1.48	93	42.87	1.56	1.18	–3.18 to ∞

b: regression coefficient, CCA: complete-case analysis, CG: control group, IG: intervention group, ITTA: intention-to-treat analysis, PPA: per-protocol analysis. Multiple imputation was used for the ITTA, non-adjusted ITTA and PPA. The non-inferiority margin is –4 points.

### Secondary outcomes

In our adjusted intention-to-treat analysis (Tables III and IV) and the adjusted per-protocol analysis (Tables SIII and SIV), there were no significant group differences at the 12-month follow-up. In our adjusted intention-to-treat analysis, however, the significance level for better subjective coping skills favouring the intervention group was only slightly exceeded (b = 1.12; 95% CI = –0.01 to 2.25; *p* = 0.051). The non-adjusted intention-to-treat analysis (b = 1.29; 95% CI = 0.12 to 2.45; *p* = 0.031) and the adjusted complete-case analysis (b = 1.52; 95% CI = 0.34 to 2.70; *p* = 0.012) each showed significantly better subjective coping competence in favour of the intervention group at the 12-month follow-up (Tables SV–SVIII).

**Table III:** Secondary continuous outcomes: adjusted intention-to-treat analysis

Item	IG (*n* = 127)		CG (*n* = 143)		b	95% CI	*p*-value*
	Predicted value	SE	Predicted value	SE			
Current health status (0–10)	6.33	0.22	5.99	0.20	0.34	–0.27–0.96	0.273
Mental health (0–10)	6.04	0.24	5.89	0.26	0.16	–0.57–0.88	0.666
Functional capacity (0–10)	6.11	0.25	5.97	0.23	0.14	–0.58–0.85	0.701
Pain (0–10)	4.64	0.25	4.76	0.20	–0.12	–0.73–0.48	0.691
Action-oriented coping (4–20)	18.08	0.47	17.62	0.41	0.46	–0.75–1.66	0.452
Cognitive restructuring (4–20)	14.95	0.46	14.35	0.47	0.60	–0.72–1.92	0.368
Subjective coping competence (4–20)	18.05	0.46	16.93	0.40	1.12	–0.01–2.25	0.051
Mental distraction (4–20)	11.62	0.53	11.53	0.57	0.09	–1.26–1.44	0.893
Counter-activities (4–20)	13.49	0.56	13.37	0.45	0.12	–1.30–1.53	0.872
Relaxation (4–20)	12.49	0.58	13.10	0.50	–0.61	–2.12–0.89	0.422
Disorder and treatment knowledge (0–50)	37.74	1.15	36.87	1.17	0.87	–2.48–4.21	0.605
Self-efficacy in practising gained knowledge (0–20)	14.15	0.54	13.84	0.44	0.31	–1.11–1.73	0.662
Electronic health literacy (8–40)	32.58	0.56	32.68	0.55	–0.09	–1.60–1.40	0.902
Work ability in relation to work demands (2–10)	7.05	0.25	6.87	0.22	0.19	–0.50–0.86	0.586
Self-rated work ability (0–10)	6.18	0.32	6.01	0.28	0.18	–0.66–1.01	0.676
Sickness absence in weeks (0–26)	4.90	1.01	5.83	1.03	–0.93	–3.98–2.12	0.543

b: regression coefficient; CG: control group; IG: intervention group.

* Linear regression. Multiple imputation was used for the adjusted intention-to-treat analysis.

**Table IV:** Secondary binary outcomes: adjusted intention-to-treat analysis

Factor	IG (*n* = 127)		CG (*n* = 143)		OR	95% CI	*p-*value*
	Predicted value	SE	Predicted value	SE			
Employment: yes^a^	0.87	0.04	0.83	0.04	1.29	0.55–3.02	0.558
Off work due to sickness absence: yes	0.21	0.05	0.22	0.05	0.94	0.40–2.19	0.878
Self-informing behaviour: at least once per week	0.15	0.05	0.18	0.05	0.80	0.31–2.08	0.646
Adherence to exercises: at least once per week	0.52	0.06	0.54	0.05	0.94	0.49–1.80	0.855
Adherence to knowledge: at least once per week	0.58	0.06	0.52	0.05	1.26	0.67–2.36	0.463

CG: control group; IG: intervention group; OR: odds ratio.

* Logistic regression.

a Due to perfect prediction of the variable, no adjustment for baseline values and outpatient rehabilitation centre. Multiple imputation was used for the adjusted intention-to-treat analysis.

### Moderator analysis

There were no significant moderating effects of age, sex, the level of education, electronic health literacy, and motivational self-efficacy (*p* > 0.05).

### Adverse effect

No adverse effects or unintended effects were reported in either group during the study.

## DISCUSSION

This randomized controlled trial evaluated the effectiveness of digitally assisted rehabilitation, which incorporated a multimodal digital back school, compared with conventional rehabilitation that delivered the same back school through in-person sessions for patients with back pain. At the 12-month follow-up, we found that digitally assisted rehabilitation was non-inferior for our primary outcome, pain self-efficacy. Our primary outcome results remained consistent across all analyses, including adjusted and non-adjusted intention-to-treat, adjusted complete case, and adjusted per-protocol analyses.

The results for our secondary outcomes showed better subjective coping competence in favour of the intervention group in our non-adjusted intention-to-treat analysis and adjusted complete-case analysis. In addition, subjective coping competence in our adjusted intention-to-treat was close to being in favour of the intervention group. In the adjusted complete-case and per-protocol analysis at the 3-month follow-up, we found better counter-activities for the intervention group (17). Both outcomes, counter-activities and coping competence, represent pain management (28). This is a subtle indication that the digital back school may be slightly superior in teaching cognitive and behavioural pain management.

Our 12-month follow-up results also showed that the non-inferiority of digitally assisted rehabilitation was maintained from the end of rehabilitation to 1 year after rehabilitation. Particularly in the case of a health condition such as back pain with the potential for recurrent episodes, it is important that the long-term effects of treatment are sustained.

The effectiveness of telerehabilitation has been investigated worldwide in numerous studies on various health problems and compared with in-person rehabilitation (9, 43–48). The results generally showed that the effectiveness of telerehabilitation was comparable to in-person rehabilitation. Given these comparable results, the choice between in-person rehabilitation and telerehabilitation could be made individually for each patient, ensuring the best possible fit for their specific needs (49). Moreover, should telerehabilitative services become a permanent part of patient care, healthcare providers will face questions about which patients are best suited for telerehabilitative services, how to maximize their effectiveness, and how to implement them most effectively in clinical practice (50). Studies on digital therapeutic interventions should therefore examine characteristics that moderate the effectiveness of these interventions in order to identify which patients are most suitable for them (50). McLaughlin et al. identified characteristics of patients with chronic back pain that could support the effectiveness of a digital intervention as part of therapy, such as employment status (part-time or full-time), high pain self-efficacy, and high scores on the Work Ability Score (50).

### Strengths and limitations

The strengths and weaknesses of this randomized controlled trial have already been described in the previous publication of the short-term results at the end of rehabilitation and the 3-month follow-up (17). We will therefore only discuss issues specifically related to the 12-month follow-up.

Although the dropout rate did not increase much compared with the 3-month follow-up, it was high in both groups, and the risk of attrition bias threatens the internal validity of our results. A non-response analysis was carried out, whereby responders and non-responders were compared with each other overall (Table SIX). We found several significant group differences at baseline, such as a higher rate of unemployment among non-responders as well as lower pain self-efficacy and a lower level of education among non-responders, which argues against the missing data being completely at random (MCAR). Instead, the differences suggest that the data were missing at random (MAR). Therefore, variables related to non-response were incorporated into the imputation model. Dropout occurred similarly in both the intervention and control groups, suggesting that missingness was not strongly differential by group. An important strength of our study was the 12-month follow-up, as most studies on digital interventions have primarily focused on short-term effects.

Although the intervention group attended the rehabilitation centre daily, participation in the digitally assisted rehabilitation program still resulted in advantages. Study participants were able to integrate the digital back school into their individual daily routines and thereby increased flexibility, for example in favour of childcare responsibilities. In addition, educational videos could be accessed repeatedly, the physical exercise videos facilitated the transfer of exercises into the home environment, and the chat function allowed participants to ask questions about the content of the digital back school at any time. We assume that digitalization is particularly suitable for aftercare. The transition from outpatient or inpatient rehabilitation to patients’ everyday lives frequently constitutes a substantial barrier. Consequently, the sustainable transfer of learned behaviours into daily routines often fails. In particular, the continuation of exercises learned during rehabilitation within one’s home environment is considered a major barrier. Rehabilitation measures monitored by therapists in the patient’s own environment can sustainably strengthen personal responsibility and help secure long-term rehabilitation success.

Future research should identify which specific components of multimodal rehabilitation can be digitized, and to what extent, without compromising its effectiveness. This could enhance the flexibility of rehabilitation. Future studies should research whether models of digitally assisted rehabilitation can be effectively applied to other patient groups and in other formats such as rehabilitation aftercare.

### Conclusion

This study provides evidence that digitally assisted rehabilitation is a viable option for patients with back pain in an outpatient setting. We were able to show that digitally assisted rehabilitation was not inferior to conventional in-person rehabilitation in the long term. This new concept of digitally assisted rehabilitation can be seen as a promising opportunity to combine the advantages of in-person rehabilitation and telerehabilitation, while maintaining non-inferior clinical effectiveness and simultaneously improving accessibility, continuity of care, and patient engagement across diverse clinical settings.

## Supplementary Material


