# Supplementary material for: NON-INFERIORITY OF DIGITALLY ASSISTED OUTPATIENT REHABILITATION IN PATIENTS WITH BACK PAIN: 12-MONTH FOLLOW-UP OF A RANDOMIZED CONTROLLED TRIAL
Source: J Rehabil Med. 2026 May 20;58:44366. doi: 10.2340/jrm.v58.44366 (PMC13196279; doi:10.2340/jrm.v58.44366)
Supplement: Supplementary file 1 [file JRM-58-44366-s1.pdf]

Supplementary material has been published as submitted. It has not been copyedited, or typeset by Journal of Rehabilitation Medicine

Table SI. Description of the intervention group based on the TIDieR checklist and the TIDieR-Telehealth checklist.

**Brief name**	Digital back school (digitally assisted rehabilitation)
**Why**	The back school aimed to increase physical activity in everyday life, according to the health action process approach, and to change cognitive patterns towards pain that lead to pain chronicity, based on the fear-avoidance beliefs model [1].
**What (materials)**	All content of the back school was digitalized and accessible online, via app or web browser, using the Caspar application. Patients used their private electronic device to participate. A study assistant within each outpatient rehabilitation center gave oral and practical instructions on correct use of the Caspar application and handed out a booklet with further information on the program, including the individual login account of the patient. The multimedia content contained educational videos and videos on physical exercises. For interactive meetings, the electronic device had to be equipped with a suitable camera. Furthermore, patients could communicate with a healthcare professional, in particular physiotherapists, individually through a chat box at any time during the week.
**What (procedures)**	The back school consisted of seven modules [2]: (1) fundamentals, (2) back health and physical activity, (3) body awareness and spine stabilization, (4) mental factors, (5) posture and movement sequences in everyday life and at work, (6) physical activity in everyday life (part 1), and (7) physical activity in everyday life (part 2).
**Who provided**	Caspar Health is a private company that provided all features of the Caspar application. The Tele-Therapie-Klinik Berlin is a telemedicine clinic and provided all healthcare professionals that guided the interactive sessions in the intervention group online, including the chat. These healthcare professionals were experienced and specialized in digital therapy and were the contact persons for questions from patients about the Caspar application. In addition, all healthcare professionals involved in the study at the Tele-Therapie-Klinik Berlin had a professional qualification in a healthcare profession (e.g., physiotherapy or occupational therapy). The same applied to the study assistants in the outpatient study centers, who informed patients for the first time about the use of the Caspar application as part of the study and handed out all information documents. Any form of assistance or help during the digital intervention was provided by the healthcare professionals or the study assistant. The Tele-Therapie-Klinik Berlin planned, organized, and scheduled all the interactive meetings.
**How**	The entire back school was implemented online, using the Caspar application. Group interactive meetings were led by a healthcare professional via camera. Non-interactive parts of the modules were performed independently by each patient.

**Where**	The patients participated in the intervention from home. The healthcare professionals operated from the buildings of Tele-Therapie-Klinik Berlin or from home.
**When and how much**	The seven modules were completed within 3 weeks. Each module required 45 minutes to complete. The patients could choose freely when to use the educational videos and the videos on physical exercises during the week. In addition, every week a 45-minute live interactive meeting was conducted online via camera and a video conference tool. Interactive meetings were scheduled during the afternoon. The participants could choose from different time slots. In addition to the online back school, patients followed their individual 3-week rehabilitation program in the outpatient rehabilitation centers. Treatments in the rehabilitation programs followed the therapy standards developed by the Federal German Pension Insurance for the rehabilitation of chronic back pain [3]. After completing the back school, patients no longer had access to the Caspar application.
**Tailoring**	No specific tailoring of the intervention to the patient was planned. Nevertheless, during the weekly interactive meeting and in the chat, patients could receive individual feedback from the healthcare professional and apply it accordingly (e.g., change a physical exercise if painful).
**Modifications**	Not applicable.
**How well**	No strategies were implemented to maintain adherence to rehabilitation. However, the healthcare professionals performing the interactive online meetings at the Tele-Therapie-Klinik Berlin documented whether patients had attended the meetings, done their physical and non-physical home exercises, executed the physical home exercises correctly or needed help, watched the educative videos for each module and used the chat function. After completion of the digitalized curriculum back school, the healthcare professional at the Tele-Therapie-Klinik Berlin informed the study assistant at the respective outpatient rehabilitation center whether the patient attended the interactive online meetings. The study assistant at the outpatient rehabilitation center documented in the standardized discharge report whether the patients had attended the respective modules or not. Subsequently, the study assistant at the outpatient rehabilitation center sent the standardized discharge reports to the researchers at the University of Lübeck. The researchers at the University of Lübeck measured and assessed therapy adherence to the back school by the number of completed modules (0 to 7 modules) per patient.

A more detailed description of the intervention can be found in the supplement of the study protocol (study protocol, additional file 2).[4]

Table SII. Description of the control group based on the TIDieR checklist.

**Brief name**	Face-to-face back school (conventional rehabilitation)
**Why**	The back school aimed to increase physical activity in everyday life, according to the health action process approach, and to change cognitive patterns towards pain which lead to pain chronicity, based on the fear-avoidance beliefs model [1].
**What (materials)**	Several materials were used during the back school, such as a patient information booklet, an LCD projector, and presentation sheets. A detailed list of required materials can be found elsewhere [2].
**What (procedures)**	The back school consisted of seven modules [2]: (1) fundamentals, (2) back health and physical activity, (3) body awareness and spine stabilization, (4) mental factors, (5) posture and movement sequences in everyday life and at work, (6) physical activity in everyday life (part 1), and (7) physical activity in everyday life (part 2).
**Who provided**	All outpatient rehabilitation centers were facilities of one rehabilitation provider (Nanz Medico GmbH & Co. KG). Each outpatient rehabilitation center provided their own experts from various fields (e.g., physicians, physiotherapists, or psychologists) to implement the back school.
**How**	All interventions were carried out in groups within the outpatient rehabilitation centers in face-to-face meetings. Outside of the meetings, patients could make use of their materials, such as the patient information booklet, in order to practice or do the exercises.
**Where**	All meetings took place in the outpatient rehabilitation center.
**When and how much**	The seven modules were completed within 3 weeks. Each module required 60 minutes to complete. In addition to the back school, the patients followed their individual 3-week rehabilitation program in the outpatient rehabilitation center. The treatments in the rehabilitation programs were in accordance with the therapy standards developed by the Federal German Pension Insurance for the rehabilitation of chronic back pain [3].
**Tailoring**	No specific tailoring of the intervention to the patients was planned. Nevertheless, during the meetings patients could receive individual feedback from the experts and apply it accordingly (e.g., change a physical exercise if painful).
**Modifications**	As all included outpatient rehabilitation centers were separate institutions, slight variations in performing the back school may have occurred.
**How well**	No strategies were implemented to maintain adherence to rehabilitation. However, the expert performing the module of the respective outpatient rehabilitation center documented in the standardized discharge report whether the patients had attended the modules or not. After completion of rehabilitation, the outpatient rehabilitation centers sent the standardized discharge reports to the researchers at the University of Lübeck. The researchers at the University of Lübeck measured and assessed therapy adherence to the back school by the number of completed modules (0 to 7 modules) per patient.

Table SIII. Secondary continuous outcomes: adjusted per-protocol analysis.

	IG (n = 82)		CG (n = 93)				
	**Predicted value**	**SE**	**Predicted value**	**SE**	**b**	**95% CI**	**p-value***
Current health status (0-10)	6.31	0.27	5.85	0.25	0.46	-0.32 to 1.23	0.244
Mental health (0-10)	6.06	0.27	5.70	0.31	0.36	-0.50 to 1.20	0.407
Functional capacity (0-10)	6.10	0.31	5.81	0.28	0.29	-0.58 to 1.17	0.504
Pain (0-10)	4.67	0.30	4.60	0.24	0.07	-0.65 to 0.78	0.845
Action-oriented coping (4-20)	17.75	0.56	17.62	0.53	0.13	-1.47 to 1.71	0.875
Cognitive restructuring (4-20)	14.63	0.54	14.34	0.57	0.30	-1.33 to 1.93	0.714
Subjective coping competence (4-20)	17.80	0.54	16.99	0.51	0.81	-0.69 to 2.29	0.285
Mental distraction (4-20)	11.32	0.61	11.62	0.69	-0.31	-1.91 to 1.29	0.704
Counter-activities (4-20)	13.15	0.62	13.51	0.55	-0.37	-2.07 to 1.33	0.669
Relaxation (4-20)	12.30	0.65	13.28	0.63	-0.98	-2.79 to 0.83	0.285
Disorder and treatment knowledge (0-50)	37.03	1.28	37.34	1.46	-0.31	-4.31 to 3.69	0.879
Self-efficacy in practicing gained knowledge (0-20)	13.96	0.56	13.86	0.56	0.10	-1.57 to 1.77	0.906
Electronic health literacy (8-40)	32.47	0.69	32.68	0.73	-0.20	-2.17 to 1.77	0.839
Work ability in relation to work demands (2-10)	7.03	0.28	6.83	0.24	0.21	-0.54 to 0.95	0.581
Self-rated work ability (0-10)	6.16	0.37	5.92	0.35	0.24	-0.81 to 1.29	0.646
Sickness absence in weeks (0-13)	5.27	1.23	6.36	1.19	-1.09	-4.66 to 2.48	0.543

Abbreviations: b = regression coefficient, CG = control group, IG = intervention group. * Linear regression. Multiple imputation was used for the adjusted per-protocol analysis.

Table SIV. Secondary binary outcomes: adjusted per-protocol analysis.

	IG (n = 82)		CG (n = 93)				
	**Predicted value**	**SE**	**Predicted value**	**SE**	**OR**	**95% CI**	**p-value***
Employment: yes[a]	0.88	0.04	0.83	0.05	1.49	0.48 to 4.63	0.488
Off work due to sickness absence: yes	0.21	0.06	0.25	0.06	0.79	0.27 to 2.34	0.671
Self-informing behavior: at least once per week	0.12	0.04	0.21	0.05	0.50	0.16 to 1.51	0.496
Adherence to exercises: at least once per week	0.51	0.08	0.56	0.06	0.79	0.35 to 1.77	0.565
Adherence to knowledge: at least once per week	0.55	0.07	0.53	0.06	1.10	0.54 to 2.25	0.789

Abbreviations: CG = control group, IG = intervention group, OR = odds ratio. * Logistic regression. [a] Due to perfect prediction of the variable, no adjustment for baseline values and outpatient rehabilitation center. Multiple imputation was used for the adjusted per-protocol analysis.

Table SV. Secondary continuous outcomes: non-adjusted intention-to-treat analysis.

	IG (n = 127)		CG (n = 143)				
	**Predicted value**	**SE**	**Predicted value**	**SE**	**b**	**95% CI**	**p***
Current health status (0-10)	6.35	0.23	5.97	0.20	0.39	-0.23 to 1.00	0.220
Mental health (0-10)	5.98	0.27	5.94	0.28	0.04	-0.76 to 0.82	0.929
Functional capacity (0-10)	6.21	0.27	5.87	0.25	0.34	-0.43 to 1.10	0.382
Pain (0-10)	4.73	0.26	4.68	0.22	0.05	-0.58 to 0.67	0.877
Action-oriented coping (4-20)	18.12	0.49	17.59	0.43	0.53	-0.72 to 1.78	0.402
Cognitive restructuring (4-20)	15.10	0.49	14.21	0.49	0.88	-0.50 to 2.26	0.207
Subjective coping competence (4-20)	18.14	0.47	16.85	0.42	1.29	0.12 to 2.45	0.031
Mental distraction (4-20)	11.52	0.57	11.62	0.59	-0.10	-1.56 to 1.35	0.893
Counter-activities (4-20)	13.57	0.57	13.30	0.46	0.26	-1.19 to 1.71	0.719
Relaxation (4-20)	12.60	0.61	13.01	0.53	-0.41	-1.99 to 1.16	0.604
Disorder and treatment knowledge (0-50)	37.56	1.18	37.04	1.19	0.51	-2.90 to 3.93	0.765
Self-efficacy in practicing gained knowledge (0-20)	14.15	0.54	13.83	0.44	0.32	-1.11 to 1.75	0.658
Electronic health literacy (8-40)	32.56	0.59	32.70	0.58	-0.14	-1.73 to 1.44	0.858
Work ability in relation to work demands (2-10)	7.05	0.26	6.87	0.24	0.18	-0.54 to 0.89	0.627
Self-rated work ability (0-10)	6.18	0.34	6.01	0.29	0.16	-0.71 to 1.04	0.710
Sickness absence in weeks (0-13)	-1.39	0.30	-1.28	0.29	0.89	0.39 to 2.03	0.787

Abbreviations: b = regression coefficient, CG = control group, IG = intervention group. * Linear regression. Multiple imputation was used for the non-adjusted intention-to-treat analysis.

Table SVI. Secondary binary outcomes: non-adjusted intention-to-treat analysis.

	IG (n = 127)		CG (n = 143)				
	**Predicted value**	**SE**	**Predicted value**	**SE**	**OR**	**95% CI**	**p***
Employment: yes	0.87	0.04	0.83	0.04	1.29	0.55 to 3.02	0.558
Off work due to sickness absence: yes	0.20	0.05	0.22	0.05	0.89	0.39 to 2.03	0.787
Self-informing behavior: at least once per week	0.15	0.05	0.18	0.05	0.80	0.31 to 2.03	0.633
Adherence to exercises: at least once per week	0.52	0.06	0.54	0.05	0.94	0.49 to 1.80	0.855
Adherence to knowledge: at least once per week	0.58	0.06	0.52	0.05	1.26	0.67 to 2.36	0.463

Abbreviations: CG = control group, IG = intervention group, OR = odds ratio. * Logistic regression. Multiple imputation was used for the non-adjusted intention-to-treat analysis.

Table SVII. Secondary continuous outcomes: adjusted complete-case analysis.

	IG			CG					
	**n**	**Predicted value**	**SE**	**n**	**Predicted value**	**SE**	**b**	**95% CI**	**p***
Current health status (0-10)	69	6.57	0.24	85	6.08	0.22	0.48	-0.16 to 1.12	0.141
Mental health (0-10)	69	6.34	0.22	84	6.04	0.20	0.30	-0.29 to 0.88	0.886
Functional capacity (0-10)	69	6.21	0.24	81	6.14	0.22	0.07	-0.59 to 0.72	0.839
Pain (0-10)	69	4.89	0.24	85	4.91	0.22	-0.01	-0.67 to 0.63	0.966
Action-oriented coping (4-20)	68	18.51	0.46	79	17.47	0.42	1.04	-0.19 to 2.27	0.097
Cognitive restructuring (4-20)	68	15.11	0.43	78	14.06	0.40	1.05	-0.11 to 2.21	0.076
Subjective coping competence (4-20)	68	18.57	0.44	79	17.05	0.40	1.52	0.34 to 2.70	0.012
Mental distraction (4-20)	69	11.63	0.47	78	11.07	0.45	0.56	-0.74 to 1.85	0.399
Counter-activities (4-20)	69	13.86	0.52	80	12.97	0.48	0.89	-0.52 to 2.29	0.214
Relaxation (4-20)	68	12.84	0.52	80	12.78	0.48	0.06	-1.34 to 1.45	0.933
Disorder and treatment knowledge (0-50)	68	39.07	1.17	82	37.19	1.07	1.88	-1.27 to 5.02	0.241
Self-efficacy in practicing gained knowledge (0-20)	68	14.74	0.49	80	14.25	0.45	0.49	-0.84 to 1.82	0.465
Electronic health literacy (8-40)	67	33.31	0.60	80	33.41	0.55	-0.10	-1.71 to 1.51	0.904
Work ability in relation to work demands (2-10)	69	7.43	0.21	80	7.10	0.19	0.33	-0.23 to 0.89	0.248
Self-rated work ability (0-10)	68	6.44	0.31	84	6.36	0.28	0.08	-0.74 to 0.90	0.842
Sickness absence in weeks (0-13)	70	0.16	0.05	81	0.19	0.04	0.85	0.34 to 2.15	0.737

Abbreviations: b = regression coefficient, CG = control group, IG = intervention group. * Linear regression.

Table SVIII. Secondary binary outcomes: adjusted complete-case analysis.

	IG			CG					
	**n**	**Predicted value**	**SE**	**n**	**Predicted value**	**SE**	**OR**	**95% CI**	**p***
Employment: yes[a]	70	0.96	0.02	81	0.89	0.03	2.79	0.72 to 10.75	0.136
Off work due to sickness absence: yes[a]	71	0.14	0.04	84	0.19	0.04	0.70	0.29 to 1.65	0.411
Self-informing behavior: at least once per week	69	0.12	0.04	80	0.12	0.04	0.97	0.35 to 2.66	0.953
Adherence to exercises: at least once per week	71	0.52	0.06	85	0.54	0.05	0.89	0.46 to 1.73	0.736
Adherence to knowledge: at least once per week	71	0.56	0.06	85	0.52	0.05	1.22	0.63 to 2.34	0.559

Abbreviations: CG = control group, IG = intervention group, OR = odds ratio. * Logistic regression. [a] Due to perfect prediction of the variable, no adjustment for baseline values and outpatient rehabilitation center.

Table SIX. Sample characteristics of the non-responders versus responders 12 months after the end of rehabilitation.

	Non-responders (n = 109)	Responders (n = 161)	p*
	M (SD) or %	M (SD) or %	
Outpatient rehabilitation center			0.666
Berlin-Center	5.3%	5.7%	
Berlin-Spandau	14.2%	11.5%	
Bielefeld	19.5%	21.7%	
Frankfurt	4.4%	5.7%	
Jena	8.0%	10.8%	
München	14.2%	7.0%	
Paderborn	15.0%	17.8%	
Regensburg	19.5%	19.7%	
Sex			0.113
Male	53.1%	43.3%	
Female	46.9%	56.7%	
Age (in years)	45.1 (10.5)	48.0 (10.1)	0.027
Native German speaker			0.541
Yes	86.7%	89.2%	
No	13.3%	10.8%	
Relationship			0.028
Yes	72.5%	83.8%	
No	27.5%	16.2%	
Number of children			0.776
None	28.6%	32.2%	
1 child	23.2%	20.4%	
2 children	33.9%	36.2%	
3 children	12.5%	8.6%	
>3 children	1.8%	2.6%	
Off work due to sickness absence			<0.001
Yes	66.4%	45.1%	
No	33.6%	54.9%	
Sickness absence (in weeks)	10.6 (9.4)	8.6 (8.7)	0.068
Employment contract			0.844
Permanent employment contract	94.4%	95.0%	
Fixed-term employment contract	5.6%	5.0%	
Shift work			0.446
No	71.4%	78.0%	
Yes. I work in 2 shifts	15.4%	13.5%	
Yes. I work in 3 shifts or more	13.2%	8.5%	
Level of education			0.061
Low	10.8%	5.2%	
Medium	59.5%	53.2%	
High	29.7%	41.6%	
Employment			0.013
Yes	83.9%	93.5%	
No	16.1%	6.5%	
Pain self-efficacy (10–60)	35.7 (11.5)	39.5 (9.8)	0.004
Current health status (0–10)	4.8 (1.8)	4.9 (1.8)	0.528
Mental health (0–10)	4.8 (2.2)	5.2 (2.3)	0.127
Functional capacity (0–10)	4.0 (2.1)	4.4 (2.0)	0.160
Pain (0–10)[a]	3.0 (1.9)	2.9 (1.5)	0.889
Action-oriented coping (4–24)	16.0 (5.1)	16.3 (4.6)	0.618
Cognitive restructuring (4–24)	13.2 (4.4)	12.7 (4.7)	0.380
Subjective coping competence (4–24)	16.3 (4.6)	16.7 (3.8)	0.509

Mental distraction (4–24)	11.6 (4.5)	10.8 (4.7)	0.155
Counter-activities (4–24)	13.0 (4.7)	12.9 (4.6)	0.866
Relaxation (4-24)	11.2 (5.0)	11.0 (4.7)	0.689
Motivational self-efficacy (3-12)	9.8 (2.1)	10.4 (1.8)	0.019
Disorder and treatment knowledge (0-50)	24.7 (12.8)	23.9 (11.4)	0.575
Self-efficacy in practicing gained knowledge (0-20)	10.1 (5.0)	10.4 (4.1)	0.630
Electronic health literacy (8-40)	28.7 (6.4)	29.8 (6.1)	0.155
Self-informing behavior			0.520
At least once a week	65.8%	69.5%	
Less than once a week	34.2%	30.5%	
Work ability in relation to work demands (2-10)	5.6 (1.8)	6.1 (1.9)	0.020
Self-rated work ability (0-10)	4.1 (2.5)	4.7 (2.5)	0.052

Abbreviation: M = mean. * Two-sample t-test or Pearson χ^2 test.

Supplementary References

1. *Meng K, Seekatz B, Roßband H, Worringen U, Faller H et al.* Entwicklung eines standardisierten Rückenschulungsprogramms für die orthopädische Rehabilitation. Rehabilitation 2009; 48: 335-344
2. *Hoppe K, Oehme J, Worringen U.* Curriculum Rückenschule. Standardisierte Patientenschulung. Berlin: Deutsche Rentenversicherung Bund; 2019
3. *Deutsche Rentenversicherung.* Reha-Therapiestandards Chronischer Rückenschmerz. Berlin: Deutsche Rentenversicherung Bund; 2020
4. *Albers R, Lemke S, Knapp S, Krischak G & Bethge, M*. Non-inferiority of a hybrid outpatient rehabilitation: a randomized controlled trial (HIRE, DRKS00028770). BMC Digit Health 2023; 1: 1-13.
